# Comparative in Vitro Evaluation of Marginal Sealing in Class I Composite Restorations Using Fifth- and Seventh-Generation Adhesives

**DOI:** 10.3390/jfb16080301

**Published:** 2025-08-20

**Authors:** Serban Talpos Niculescu, Ioana Veja, George-Dumitru Constantin, Ioana Elena Lile, Christos Armeniakos, Ioana Roxana Munteanu, Tareq Hajaj

**Affiliations:** 1 Department of Oral and Maxillofacial Surgery, Faculty of Dentistry, Victor Babes University of Medicine and Pharmacy, 2 Eftimie Murgu Sq., 300041 Timisoara, Romania; talpos.serban@umft.ro; 2 Department of Dental Medicine, Faculty of Dentistry, “Vasile Goldis” Western University of Arad, Str. Liviu Rebreanu 86, 310045 Arad, Romania; veja.ioana@uvvg.ro; 3 Discipline of Clinical Practical Skills, Department I Nursing, Faculty of Medicine, Victor Babes University of Medicine and Pharmacy, 300041 Timisoara, Romania; 4 Department of Prosthodontics, Aristotle University of Thessaloniki, 541 24 Thessaloniki, Greece; carmeni@dent.auth.gr; 5 Department of Oral Rehabilitation and Dental Emergencies, Faculty of Dentistry, Victor Babes University of Medicine and Pharmacy, Eftimie Murgu Square No. 2, 300041 Timisoara, Romania; munteanu.roxana@umft.ro; 6 Interdisciplinary Research Center for Dental Medical Research, Lasers and Innovative Technologies, Revolutiei 1989 Avenue No. 9, 300070 Timisoara, Romania; 7 Department of Prostheses Technology and Dental Materials, Faculty of Dentistry, Victor Babes University of Medicine and Pharmacy, 2 Eftimie Murgu Sq., 300041 Timisoara, Romania; tareq.hajaj@umft.ro; 8 Research Center in Dental Medicine Using Conventional and Alternative Technologies, Faculty of Dental Medicine, Victor Babes University of Medicine and Pharmacy of Timisoara, 9 Revolutiei 1989 Ave, 300070 Timisoara, Romania

**Keywords:** microleakage, dental adhesives, total etch, self-etch, composite restoration, bonding agents, dye penetration, class I cavity, adhesive systems, tooth–restoration interface

## Abstract

Background: A major challenge in adhesive dentistry, often leading to restoration failure, is microleakage. This in vitro comparative study was designed to assess microleakage at the tooth–composite interface. The investigation aimed to compare the sealing efficacy of two commonly used adhesive systems. Methods: Standardized Class I cavities were prepared on 20 extracted human molars and randomly divided into two groups (n = 10 each). Group A was treated with a fifth-generation total-etch adhesive (OptiBond™ Solo Plus, Kerr Corporation, Orange, CA, USA), and Group B received a seventh-generation self-etch adhesive (Adhese® Universal VivaPen®, Ivoclar Vivadent AG, Schaan, Liechtenstein). All restorations were completed using Herculite XRV composite resin. Microleakage was evaluated using dye penetration analysis after immersion in 2% methylene blue for 10 days, followed by longitudinal sectioning and microscopic measurement at 500× magnification. Results: The fifth-generation adhesive group showed a mean microleakage of 0.2503 ± 0.1921 mm, while the seventh-generation group recorded 0.2484 ± 0.1764 mm. Statistical analysis using an independent t-test revealed no significant difference between the groups (*p* = 0.696). Conclusions: Both adhesive systems demonstrated comparable performance in minimizing microleakage under standardized conditions. Although the total-etch group exhibited slightly lower numerical values, the difference was not statistically significant. These findings suggest that both adhesive approaches can be clinically effective when applied appropriately.

## 1. Introduction

Dental caries remain one of the most prevalent chronic diseases worldwide, affecting individuals across all age groups despite ongoing advances in preventive strategies [1]. As a consequence, restorative interventions continue to be central in modern dental practice. Resin-based composite restorations, particularly for occlusal surfaces (Class I cavities), are routinely used due to their esthetic appeal and adhesive capabilities. However, a critical factor influencing the longevity and success of these restorations is the ability of adhesive systems to achieve a durable seal at the tooth–composite interface. Microleakage at this interface remains a major concern, as it can lead to postoperative sensitivity, marginal discoloration, secondary caries, and restoration failure. Given the continuous evolution of adhesive technologies—from multi-step total-etch to simplified self-etch systems—comparative investigations are essential to guide evidence-based clinical decision-making. This study aims to address this need by evaluating the sealing efficacy of two commonly used adhesive generations under controlled in vitro conditions.

Class I cavities, as defined by G.V. Black, are confined to the pits and fissures of posterior teeth and are among the most frequently encountered lesions in clinical practice [2]. These lesions are commonly restored with resin-based composites due to their excellent esthetic properties, conservative tooth preparation, and ability to bond micromechanically and chemically to dental hard tissues [3]. Nevertheless, the longevity and clinical success of these restorations depend significantly on the quality and durability of the bond formed between the tooth substrate and the restorative material. Class I occlusal cavities were chosen for this study due to their enamel-only margins, which allow for consistent substrate characteristics and reduced variability in bonding outcomes. Although considered less clinically demanding than restorations involving dentin or cementum, Class I cavities present their own challenge through a high configuration factor (C-factor), which can intensify polymerization shrinkage stress at the adhesive interface [4,5]. This model thus provides a clinically relevant yet controlled scenario for comparing the intrinsic performance of different adhesive systems, minimizing the influence of anatomical or substrate-related confounding factors.

Fifth- and seventh-generation adhesives were selected for this comparative evaluation due to their widespread clinical use and fundamentally different bonding mechanisms. Fifth-generation systems (etch-and-rinse) are well established for their strong and predictable enamel bonding through micromechanical retention, while seventh-generation adhesives (all-in-one self-etch) offer the advantage of simplified application and reduced technique sensitivity. Despite the emergence of newer universal adhesives, these two generations remain relevant in daily practice, especially in settings where cost, procedural time, or operator experience may influence adhesive selection. Moreover, conflicting reports in the literature regarding their relative performance in enamel-only restorations underscore the need for direct comparison under standardized conditions [6,7,8,9,10,11,12].

One of the most persistent challenges in adhesive dentistry is microleakage—defined as the microscopic ingress of bacteria, fluids, and chemical substances at the tooth–restoration interface. This phenomenon can compromise pulpal health and lead to postoperative sensitivity, marginal discoloration, secondary caries, and ultimately restoration failure [13,14]. Achieving a durable and effective marginal seal is, therefore, paramount for the long-term success of adhesive restorations.

Several interrelated factors influence the occurrence of microleakage, including the material’s polymerization shrinkage, the configuration factor (C-factor) of the cavity, thermal and mechanical stress, mismatched expansion coefficients, adhesive viscosity, and the clinician’s technique sensitivity [8,15]. Among these, polymerization shrinkage is particularly critical; the resulting contraction stresses can exceed 13–17 MPa, potentially disrupting the adhesive interface and inducing marginal gaps [5].

In response to these challenges, adhesive systems have undergone significant evolution over multiple generations. Fifth-generation adhesives, known as total-etch or etch-and-rinse systems, require separate phosphoric acid etching followed by the application of a combined primer and adhesive. These systems are valued for their predictable enamel bonding and the formation of a well-defined hybrid layer in dentin [6]. Seventh-generation adhesives, or self-etch systems, streamline the procedure by incorporating the etchant, primer, and bonding agent into a single-step application, thereby reducing clinical time and minimizing operator variability [7]. However, concerns have been raised about the limited etching capacity of self-etch adhesives on uncut enamel due to their lower acidity, which may result in weaker enamel bonding [8,9].

Contemporary literature provides conflicting evidence regarding the clinical and laboratory performance of these adhesive strategies. Some studies report comparable outcomes between total-etch and self-etch systems, particularly in enamel-dominant restorations [10,16], while others emphasize the superior sealing ability of total-etch techniques under complex conditions such as dentin margins, aging, or high-C-factor cavities [11,12]. These discrepancies underscore the need for further controlled investigations focused on standard cavity configurations.

Dye penetration testing remains one of the most frequently employed methods for assessing microleakage in vitro. While this approach is inherently destructive and limited in spatial resolution, it is valued for its affordability, simplicity, and sensitivity, offering reliable comparative data on adhesive performance [17].

Despite continuous advancements—including the development of universal adhesives capable of multiple application modes, the addition of nanofillers, and improved solvent systems—there is still no consensus on the relative efficacy of traditional total-etch versus modern self-etch systems in achieving marginal sealing in Class I enamel-only restorations. Given that enamel bonding is critical to preventing microleakage, particularly in occlusal restorations, further investigation is warranted using controlled in vitro models.

The aim of this in vitro study was to compare the marginal sealing ability of a fifth-generation total-etch adhesive (OptiBond™ Solo Plus, Kerr Corporation, Orange, CA, USA) and a seventh-generation self-etch adhesive (Adhese® Universal VivaPen®, Ivoclar Vivadent AG, Schaan, Liechtenstein) in standardized Class I composite restorations. The primary objective was to quantitatively assess and compare the degree of microleakage at the tooth–composite interface using dye penetration analysis under controlled laboratory conditions.

**Hypothesis H1:**  *The null hypothesis was that there would be no statistically significant difference in microleakage between the two adhesive systems.*

## 2. Materials and Methods

### 2.1. Sample Collection and Cavity Preparation

Twenty freshly extracted human third molars were collected for this in vitro study. Teeth were extracted for non-pathological reasons (e.g., orthodontic or periodontal indications) with informed consent, in accordance with the protocol approved by the Ethics Committee of “Victor Babeș” University of Medicine and Pharmacy (approval no. 78/08.01.2024 and date of approval 8 January 2024).

Immediately post-extraction, all teeth were cleaned of soft tissue remnants and disinfected in 0.5% chloramine-T solution for 24 hours. Subsequently, they were stored in 0.9% isotonic saline at room temperature to maintain hydration until use.

Standardized Class I occlusal cavities were prepared using a high-speed turbine with water cooling for enamel removal and a low-speed contra-angle handpiece for dentin refinement, aiming to prevent thermal damage and preserve structural integrity. Each cavity measured approximately 3 mm buccolingually, 2 mm mesiodistally, and 2 mm in depth, verified with a digital caliper. This configuration corresponds to a high C-factor (approximately 5), which is known to intensify polymerization stress and potentially compromise marginal sealing. While Class I cavities are generally considered less complex due to their enamel-limited margins, their high C-factor presents a relevant and reproducible model for evaluating adhesive performance under shrinkage-induced stress.

The following rotary burs were used for cavity preparation:

- ISO 801 round bur (coarse, green)—initial outline;
- Football-shaped coarse green bur—occlusal leveling;
- Tapered conical coarse green bur—internal wall refinement;
- RA3 tungsten–carbide finishing bur—dentin floor smoothing.

Each bur was replaced after every five preparations to ensure cutting efficiency and avoid variability due to bur wear. All preparations were completed by the same calibrated operator to reduce procedural inconsistencies.

### 2.2. Group Allocation and Adhesive Systems

The prepared specimens were randomly divided into two equal groups (n = 10 each), using a computerized randomization protocol.

**Group A** (Fifth-Generation Total-Etch Adhesive): OptiBond™ Solo Plus (Kerr Corporation, Orange, CA, USA) was used in conjunction with OptiBond™ Gel Etchant (37.5% phosphoric acid, Kerr Corporation, Orange, CA, USA). OptiBond Solo Plus is a light-cured, ethanol- and water-based adhesive containing monomers such as Bis-GMA, HEMA, and GPDM (glycerol phosphate dimethacrylate), designed for total-etch applications.

**Group B** (Seventh-Generation Self-Etch Adhesive): Adhese® Universal VivaPen® (Ivoclar Vivadent AG, Schaan, Liechtenstein) was used in self-etch mode. This light-cured adhesive contains monomers including MDP (10-Methacryloyloxydecyl dihydrogen phosphate), Bis-GMA, HEMA, and ethanol as a solvent, and is compatible with all etching techniques. The VivaPen delivery system ensures controlled intraoral dispensing and reduced material waste.

In **Group A** (fifth-generation, total-etch adhesive), the bonding procedure began with the application of 37.5% phosphoric acid gel to the enamel and dentin surfaces for 15 s. The etchant was then thoroughly rinsed off with water for 10 seconds, and the cavity was gently air-dried to maintain a moist surface, in accordance with the wet bonding technique. Following this, OptiBond™ Solo Plus adhesive was applied using a microbrush in a brushing motion for 15 seconds to ensure proper infiltration. The adhesive layer was then air-thinned for 3 seconds to achieve uniform film thickness and promote solvent evaporation. Light polymerization was carried out for 20 seconds using an Elipar™ DeepCure-S LED curing unit (3M ESPE, St. Paul, MN, USA), delivering an output of 1000 mW/cm^2^, with light intensity periodically verified using a Bluephase Meter II (Ivoclar Vivadent) radiometer.

In **Group B** (seventh-generation, self-etch adhesive), the Adhese® Universal VivaPen® was used in self-etch mode, without prior phosphoric acid conditioning. The adhesive was dispensed directly into the cavity and actively scrubbed into the surface for 20 seconds to enhance chemical interaction with the tooth substrate. This was followed by a gentle air stream to create a uniform, glossy, and immobile adhesive film. Polymerization was performed under identical conditions to Group A, using the same LED curing unit and light intensity parameters.

All bonding procedures were carried out in a controlled environment with constant temperature (23 ± 1°C) and relative humidity (50 ± 5%), adhering strictly to the manufacturers’ recommended protocols. All bonding procedures were performed by the same calibrated operator who prepared the cavities, in order to minimize operator-dependent variability.

### 2.3. Restoration Procedure

All cavities were restored with a single increment of Herculite XRV Enamel composite resin (Kerr Corporation, Orange, CA, USA), a microhybrid light-cured material. Adaptation was achieved with a plastic filling instrument, and polymerization was performed using the Elipar™ DeepCure-S LED unit (3M ESPE, St. Paul, MN, USA) for 40 seconds at ~1 mm distance.

Curing output was monitored periodically with a Bluephase Meter II (Ivoclar Vivadent AG, Schaan, Liechtenstein) to ensure consistent light intensity. After curing, specimens were stored in 0.9% saline at 37 °C for 24 hours to allow post-curing stress relief. All specimens were visually inspected under magnification to confirm the absence of marginal defects before dye immersion. Composite placement and light curing were likewise completed by the same operator under standardized conditions.

### 2.4. Dye Penetration and Microleakage Assessment

Specimens were individually immersed in 0.9 mL of 2% methylene blue solution (Sigma-Aldrich, St. Louis, MO, USA) for 10 days in light-proof containers, with dye refreshed every 48 hours. After immersion, teeth were rinsed and longitudinally sectioned mesiodistally using a water-cooled low-speed diamond saw (Isomet® 1000, Buehler Ltd., Lake Bluff, IL, USA).

Images were captured at 500× magnification using the CoolingTech USB Digital Microscope (Model G600, Shenzhen Cooling Technology Co., Ltd., Shenzhen, China), and analyzed with AmScope Measurement Software v4.11 (United Scope LLC, Irvine, CA, USA).

Two independent, blinded examiners performed all measurements. The inter-examiner agreement was assessed by calculating the intraclass correlation coefficient (ICC) using a two-way random-effects model with absolute agreement. In cases of discrepancies > 0.02 mm, a consensus value was established.

Microleakage was assessed at the occlusal cavosurface margins of standardized Class I restorations. Because the cavity preparations were confined to occlusal pits and fissures, all margins were located in enamel, with no involvement of gingival or cervical interfaces. Dye penetration was measured linearly from the external enamel margin inward along the tooth–composite interface.

Microleakage was evaluated using the dye penetration technique, a standard method based on the ability of low-molecular-weight dyes to infiltrate microscopic gaps at the tooth–restoration interface. After 24 hours of saline storage, each specimen was individually immersed in 0.9 mL of freshly prepared 2% methylene blue solution (pH ~7.0) for 10 days at room temperature in light-proof containers. The dye was refreshed every 48 hours to maintain staining efficacy.

Following immersion, the teeth were rinsed under running distilled water for 1 min and gently dried. Longitudinal sectioning in the mesiodistal plane was performed using a water-cooled low-speed diamond saw (Isomet® 1000, Buehler Ltd., Lake Bluff, IL, USA) to expose the tooth–composite interface. Each section was then examined under a digital stereomicroscope (CoolingTech USB Microscope, 50–500× magnification) at 500× magnification.

The maximum depth of dye penetration (in mm) was measured from the external enamel margin toward the pulpal floor using calibrated imaging software (AmScope Measurement Software, v4.11). Only linear dye progression at the interface was recorded. Internal staining or dye diffusion not originating from the margin was excluded.

All measurements were independently performed by two blinded examiners. Inter-examiner agreement was assessed via the intraclass correlation coefficient (ICC), and discrepancies greater than 0.02 mm were resolved by consensus.

To ensure uniform sectioning across all specimens, teeth were embedded in autopolymerizing acrylic resin blocks with the occlusal surface oriented perpendicular to the base, using a custom-made positioning jig. This orientation allowed for precise mesiodistal sectioning through the center of the restoration using a water-cooled low-speed diamond saw. Although the natural anatomy of occlusal cavities includes curved internal surfaces, all sectioning was performed along a consistent anatomical axis to minimize variability in exposure of the restoration margins. Section planes were verified under magnification to confirm passage through the central fissure and the deepest part of the cavity floor. To control the sectional plane and ensure parallel orientation relative to the long axis of the tooth and the restoration, each specimen was embedded in a standardized acrylic mold using a custom-fabricated positioning jig. The occlusal surface was aligned perpendicular to the base of the mold, and the mesiodistal axis was visually centered. Sectioning was performed using a water-cooled low-speed diamond saw (Isomet® 1000, Buehler Ltd.) with the blade aligned to the midpoint of the restoration, guided by external anatomical landmarks. Pilot cuts were verified under magnification to confirm trajectory before full sectioning. This approach minimized oblique or non-uniform slices that could compromise microleakage measurements and ensured consistent exposure of the restoration interface.

Although microleakage values were ultimately reported in millimeters, measurements were performed using calibrated digital imaging software (AmScope v4.11), with precision up to three decimal places (0.001 mm). This allowed detection of subtle differences in dye penetration, enhancing the sensitivity of the analysis despite rounding values for reporting clarity. All values were recorded to the nearest 0.001 mm and only rounded during final data presentation.

### 2.5. Statistical Analysis and Sample Size Calculation

Data were analyzed using MedCalc® version 23.0.6 (MedCalc Software Ltd., Ostend, Belgium). Normality of distribution was verified using the Shapiro–Wilk test. As data were normally distributed, an independent samples t-test was applied to compare mean microleakage values between groups. Statistical significance was set at *p* < 0.05.

Sample size was calculated using G*Power 3.1.9.7 software (Heinrich Heine University Düsseldorf, Düsseldorf, Germany), assuming a two-tailed independent samples t-test, medium effect size (Cohen’s d = 0.8), alpha = 0.05, and power = 0.80. This yielded a required minimum of 10 specimens per group, which was fulfilled in the present study. Although appropriate for detecting medium-to-large differences, this sample size may be underpowered for identifying small yet clinically relevant effects.

## 3. Results

**Figure 1:**
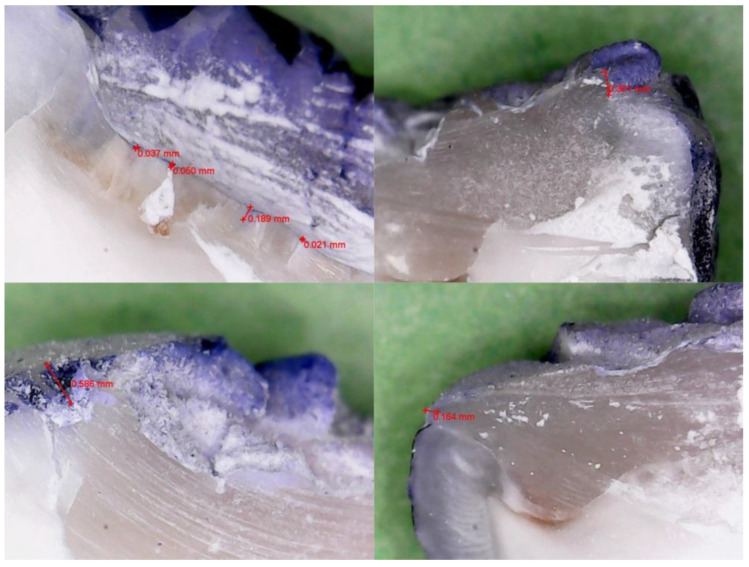
Representative sectioned specimen from the fifth-generation group (OptiBond™ Solo Plus), showing limited dye penetration at the enamel–composite interface under 500× magnification.

**Figure 2:**
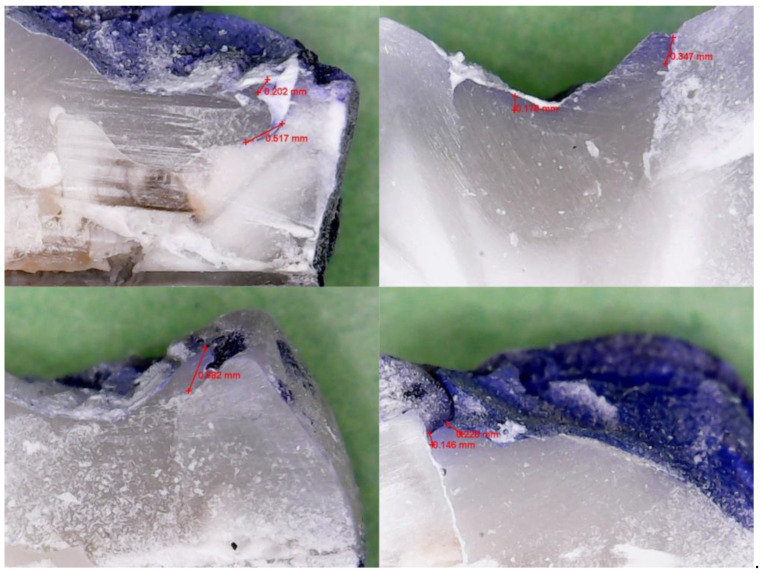
Representative sectioned specimen from the seventh-generation group (Adhese® Universal VivaPen®), illustrating comparable dye penetration under the same magnification.

Figure 1 and Figure 2 present representative samples from each group, selected to illustrate typical microleakage patterns at the tooth–composite interface. These images reflect the general distribution observed across specimens and support the quantitative findings reported.

Microleakage was evaluated in all twenty specimens, equally distributed between the two adhesive groups (n = 10 each). The extent of dye penetration at the tooth–composite interface was recorded for each sample in millimeters, as measured under 500× magnification using calibrated digital imaging. All dye penetration measurements were recorded at the occlusal margin of the Class I cavity, where the composite restoration met the enamel surface. No dentinal or cervical margins were present in the standardized cavity design.

The group restored with the fifth-generation total-etch adhesive (OptiBond™ Solo Plus) showed a mean microleakage value of 0.2503 ± 0.1921 mm, while the seventh-generation self-etch group (Adhese® Universal VivaPen®) exhibited a mean value of 0.2484 ± 0.1764 mm.

Normal distribution of the data was confirmed using the Shapiro–Wilk test (*p* > 0.05), allowing for parametric analysis. An independent samples t-test revealed no statistically significant difference between the two groups (t = −0.404, *p* = 0.696), indicating that both adhesive systems provided comparable marginal sealing under the conditions tested.

The intraclass correlation coefficient (ICC) between the two blinded examiners was 0.985 (95% CI: 0.964–0.995), indicating excellent inter-rater reliability.

Table 1 presents the individual microleakage values for each tooth, along with the calculated means and standard deviations for each adhesive system. 

The line graph with a filled area illustrates the distribution of maximum dye penetration depths across individual specimens (Tooth #1–10) for both adhesive systems. Although both groups exhibit variability, the seventh-generation adhesive (red) demonstrates slightly higher microleakage values in several samples (e.g., Tooth #1, #5, and #6), while the fifth-generation adhesive (blue) shows higher leakage in Tooth #7. Overall, the two curves are largely overlapping, supporting the statistical finding of no significant difference (*p* = 0.696). The shaded regions emphasize relative performance and intra-group dispersion, reinforcing that both adhesives provided comparable marginal sealing under standardized conditions.

## 4. Discussion

This in vitro study compared the marginal sealing performance of two adhesive strategies—a fifth-generation total-etch system and a seventh-generation self-etch system—in standardized Class I composite restorations. Although the total-etch group exhibited slightly lower numerical microleakage values, the difference was not statistically significant (*p* = 0.696), supporting the null hypothesis. Under controlled conditions and when applied as recommended, both systems demonstrated comparable sealing efficacy.

The novelty of this study lies in its controlled comparison of fifth- and seventh-generation adhesive systems under standardized, enamel-only Class I conditions. By using freshly extracted human teeth, standardized cavity dimensions, a high configuration factor (C-factor), and enamel-only margins, we aimed to isolate the sealing performance of each adhesive without the added variability introduced by dentin or cementum substrates. This design addresses an underexplored gap in the literature, where most previous studies have assessed mixed-margin restorations or combined multiple cavity classes and adhesive types in a single analysis.

The present results are consistent with prior studies showing no significant differences between total-etch and self-etch adhesives in enamel-dominant restorations [8,16]. This outcome can be attributed to the predictable bonding behavior of enamel, which enables effective micromechanical retention when proper surface preparation and adhesive protocols are followed [6,7].

Although phosphoric acid etching is widely regarded as the gold standard for optimal enamel bonding due to its ability to produce a high-energy, micro-retentive surface, the lack of significant difference in microleakage between the total-etch and self-etch groups in this study may be explained by several factors. First, the enamel in freshly extracted, non-aged teeth presents a relatively homogenous and receptive surface for bonding—even for weaker acids. The Adhese® Universal VivaPen® contains functional monomers like MDP and utilizes ethanol as a solvent, both of which contribute to effective enamel infiltration and hybridization, even without phosphoric acid pre-treatment [7,9,18]. Additionally, under strictly controlled laboratory conditions with proper adhesive handling, light curing, and single-operator application, the influence of technique sensitivity is minimized, allowing both systems to perform near their optimal capacity. This may result in performance convergence not always seen in clinical conditions, where variables such as moisture control, saliva contamination, or access to cavity margins can influence outcomes.

The comparable microleakage values observed between the fifth- and seventh-generation adhesives can be attributed to several interacting factors. First, the enamel-only margins of the standardized Class I cavities provided a substrate where both micromechanical (total-etch) and chemical (self-etch) bonding strategies could perform effectively. While total-etch adhesives like OptiBond™ Solo Plus create deep resin tags through phosphoric acid etching, self-etch systems such as Adhese® Universal rely on functional monomers (e.g., MDP) to promote chemical interaction with hydroxyapatite [7,9,18]. These interactions may have compensated for the lower etching aggressiveness of the self-etch system, particularly in enamel-dominant conditions.

Previous studies have shown inconsistent results when comparing adhesive generations, largely due to differences in cavity configuration, substrate type (enamel vs. dentin), and test conditions [10,11,12,19]. Our findings align with reports where enamel bonding was predominant and protocol standardization reduced confounding factors. However, other investigations have reported inferior sealing with self-etch adhesives in mixed-margin or dentin-based restorations, where the smear layer and hydrophilic substrate limit chemical infiltration and bonding strength [20,21].

Furthermore, the high configuration factor (C-factor) of the Class I cavities increased the potential for polymerization shrinkage stress. Despite this, both adhesives maintained marginal integrity, likely aided by the use of a microhybrid composite with low shrinkage and the single-increment placement technique. Studies have shown that incremental filling and controlled light curing can reduce polymerization stress and mitigate adhesive debonding [3,4,13].

The literature presents mixed findings regarding the relative enamel bond strength of total-etch and self-etch adhesives. Several studies have consistently reported superior bonding to uncut enamel with phosphoric acid etching due to more pronounced micromechanical retention [6,10,12]. In contrast, other reports—particularly those involving modern self-etch systems containing MDP and optimized solvent systems—have demonstrated comparable performance, especially when enamel surfaces are freshly prepared and surface energy is elevated [7,9,18,19]. These discrepancies can often be attributed to variations in substrate conditions (e.g., cut vs. uncut enamel), aging protocols, adhesive composition, operator technique, and evaluation methods (e.g., microtensile vs. microleakage testing). Our findings align with the latter group of studies and suggest that, under ideal conditions, self-etch systems may achieve satisfactory enamel sealing comparable to conventional total-etch adhesives.

Clinically, the findings suggest that with proper application and when used in enamel-only restorations, simplified self-etch adhesives may offer performance comparable to traditional total-etch systems. This could support their use in pediatric or general practice settings where time efficiency and ease of use are critical. However, caution remains necessary in more complex clinical situations where dentin exposure or deep margins are involved.

Several in vitro investigations have reported similar findings in Class I restorations using adhesives from different generations [10,16], while others noted superior sealing performance with total-etch systems, particularly under challenging substrates or aging conditions [11,12]. In our study, the standardized enamel-only configuration minimized substrate-related variability and allowed for a focused evaluation of adhesive performance.

Modern self-etch adhesives incorporate functional monomers such as MDP, which promote chemical interaction with hydroxyapatite and enhance bonding efficiency [7,9,18]. These chemical interactions may compensate for the lower etching capacity of self-etch systems, contributing to their increasingly reliable clinical outcomes.

Microleakage was assessed using dye penetration, a widely accepted, reproducible, and cost-effective method for marginal sealing evaluation [17]. Although this method lacks the resolution of advanced imaging techniques like micro-CT or confocal microscopy [22], its simplicity and sensitivity remain valuable in comparative adhesive research.

While adhesive performance is the primary factor in marginal sealing, certain material properties of the composite resin may indirectly influence microleakage outcomes. Herculite XRV, the microhybrid composite used in this study, features a relatively high filler content and low polymerization shrinkage, which may contribute to more favorable stress distribution during curing. Studies have shown that composites with lower volumetric shrinkage and higher elastic modulus tend to reduce gap formation at the adhesive interface, particularly in high-C-factor cavities [3,4,13,22]. However, it is important to note that the adhesive system remains the principal determinant of interfacial sealing, and the restorative material plays a supportive rather than primary role.

Other studies have shown increased microleakage in self-etch systems when used in restorations involving dentin or cementum margins, particularly in Class II and Class V cavities [9,11,19]. These results highlight the substrate-dependent nature of adhesive performance and the need for case-specific adhesive selection.

In vitro evaluations have also confirmed comparable performance between different generations of adhesives [20,21], while ongoing developments in adhesive formulations continue to enhance their sealing capabilities [18,22,23].

Survey-based research further contextualizes these findings, indicating a clear preference among practitioners—especially younger clinicians—for simplified, universal adhesives due to their efficiency and ease of application [24]. This trend corresponds to the practical advantages observed in the self-etch group of our study, where reduced technique sensitivity may facilitate consistent outcomes.

From a materials science standpoint, recent analyses using energy-dispersive X-ray spectroscopy have highlighted the importance of chemical interactions at the dentin–adhesive interface, reinforcing the role of interfacial stability in long-term clinical success [25].

Moreover, universal adhesives have shown promising results across various application modes. Studies have demonstrated that they maintain comparable sealing to total-etch systems even after thermomechanical aging [26], and systematic reviews confirm their versatility and long-term bonding performance [27].

The performance of adhesive systems in preventing microleakage is strongly influenced by their chemical composition, solvent system, and viscosity. OptiBond™ Solo Plus, a fifth-generation etch-and-rinse adhesive, contains Bis-GMA, GPDM, and HEMA monomers in an ethanol–water solvent base. Its relatively higher viscosity and requirement for a moist bonding protocol promote the formation of a well-defined hybrid layer but make it more technique-sensitive, particularly in cases of over-drying or inconsistent application [6,7,16].

In contrast, Adhese® Universal VivaPen® is a one-step self-etch adhesive that incorporates the functional monomer MDP, which forms stable ionic bonds with hydroxyapatite, contributing to long-term interfacial stability [12,22,27]. Its ethanol-based solvent promotes effective monomer penetration and evaporation, while the VivaPen delivery system enhances application uniformity and reduces waste. Despite its lower acidity compared to phosphoric acid, Adhese® Universal has demonstrated acceptable enamel infiltration and bonding performance in vitro, especially when applied using an active scrubbing technique [7,9,27].

These compositional differences may explain the similar performance observed in this study. The total-etch system relies primarily on micromechanical interlocking via phosphoric acid conditioning, whereas the self-etch system supplements mechanical retention with chemical adhesion. Under controlled laboratory conditions, this dual mechanism may allow self-etch adhesives to achieve marginal sealing comparable to conventional multi-step systems [8,10,18].

While the findings of this study are promising, several limitations should be acknowledged. The experimental protocol did not include thermomechanical aging, such as thermocycling or cyclic occlusal loading, which are essential to simulate the dynamic conditions of the oral environment and assess long-term restoration performance. Moreover, the use of standardized Class I cavities with enamel-only margins does not fully reflect the complexity of clinical situations involving dentin, cementum, or subgingival margins. These methodological constraints may limit the generalizability of the results to real-life clinical scenarios and long-term outcomes.

A key limitation of this study is the absence of aging protocols such as thermocycling or mechanical loading, which are essential to simulate intraoral thermal fluctuations and functional stresses. These factors may significantly impact adhesive durability and marginal adaptation over time. The decision to omit usage simulation was based on our intention to first evaluate the intrinsic sealing capacity of the two adhesive systems under ideal, controlled conditions. However, future studies should incorporate thermomechanical aging to enhance clinical relevance and assess long-term performance under conditions that more closely approximate the oral environment. 

Additionally, the relatively small sample size, although statistically justified, may have limited our ability to detect subtle differences in microleakage between the two adhesive systems. The observed variability in individual measurements could partly reflect biological heterogeneity or technique sensitivity, and future studies should consider larger sample sizes to improve statistical resolution and generalizability.

Furthermore, the prolonged storage of extracted teeth in isotonic saline, although necessary to maintain hydration, may not fully replicate the complex in vivo environment of dentin and enamel. This could potentially affect tissue permeability and bonding behavior. Additionally, the light-curing protocol involved occlusal-only irradiation, which, while clinically relevant, may not account for the potential benefits of multi-directional curing. The direction of light application could influence polymerization kinetics and shrinkage vectors, thereby affecting marginal adaptation—particularly at the line angles or cavity walls less directly exposed to curing light. These aspects should be considered in future experimental designs aiming to better simulate clinical conditions.

Nevertheless, the standardization of cavity preparation, the use of a calibrated operator, and the high inter-examiner agreement (ICC = 0.985) support the reliability of the present results. Future research should integrate aging protocols and multimodal leakage assessment to better reflect clinical performance.

Although no statistically significant differences were found between the fifth- and seventh-generation adhesives in terms of microleakage under standardized in vitro conditions, their clinical behavior may diverge over time. Fifth-generation (etch-and-rinse) systems are well-validated for long-term performance, especially in enamel bonding, but are more technique-sensitive and moisture-dependent [6,10].

In contrast, seventh-generation self-etch adhesives like Adhese® Universal offer simplified protocols and are less susceptible to operator variability. Their use of MDP enables durable chemical adhesion, which may help maintain marginal seal over time, even after thermomechanical aging [18,26,27,28]. These findings suggest that both systems can be clinically acceptable in enamel-only restorations, with selection based on case-specific requirements and operator preference.

## 5. Conclusions

Within the limitations of this in vitro study, both the fifth-generation total-etch adhesive (OptiBond™ Solo Plus) and the seventh-generation self-etch adhesive (Adhese® Universal VivaPen®) demonstrated comparable marginal sealing in standardized Class I composite restorations with enamel-only margins. Although the total-etch group showed slightly lower microleakage values, the difference was not statistically significant.

These findings suggest that, under ideal application conditions and when used according to manufacturers’ instructions, both adhesive strategies can provide effective sealing at the tooth–composite interface. In clinical practice, total-etch systems may be preferred in cases where maximum enamel bond strength is required, such as in large occlusal restorations or esthetic zones, while self-etch or universal adhesives may offer practical advantages in time-sensitive procedures or when operator variability is a concern.

Further research involving thermomechanical aging, dentin or mixed-margin restorations, and different adhesive application modes is warranted to evaluate long-term performance and guide evidence-based adhesive selection in diverse clinical scenarios. This contributes to the growing body of evidence supporting the use of universal adhesives in enamel-based restorations. These results support the clinical acceptability of both adhesive systems in Class I enamel-limited restorations, provided they are applied according to the manufacturers’ instructions. In practice, the choice between total-etch and self-etch protocols should consider clinical context, operator experience, and the need for procedural efficiency.

Additionally, continued research into the incorporation of functional additives—such as bioactive glass, calcium phosphate compounds, or antibacterial monomers—into adhesives and composites is warranted. These materials show promise in enhancing bond strength, promoting remineralization, and reducing bacterial colonization at the restoration margins. Such innovations align with the growing emphasis on biomimetic strategies aimed at improving long-term restoration stability and biological compatibility.

## Figures and Tables

**Table 1:** Microleakage values (mm) for each specimen in the fifth-generation and seventh-generation adhesive groups, along with group mean and standard deviation.

Tooth	5th Gen Adhesive (mm)	7th Gen Adhesive (mm)
1	0.586	0.517
2	0.185	0.448
3	0.092	0.089
4	0.189	0.206
5	0.109	0.582
6	0.054	0.347
7	0.593	0.146
8	0.301	0.187
9	0.230	0.146
10	0.164	0.163
Mean ± SD	0.2503 ± 0.1921	0.2484 ± 0.1764

**Note:** Microleakage values represent maximum dye penetration depth (in millimeters) at the tooth–composite interface, as measured on mesiodistal sections at 500× magnification. SD = standard deviation, calculated based on n = 10 specimens per group. No statistically significant difference was observed between the groups (independent *t*-test, *p* = 0.696).

## Data Availability

The original contributions presented in the study are included in the article; further inquiries can be directed to the corresponding authors.

## Abbreviations

The following abbreviations are used in this manuscript:

C-Factor	Configuration Factor
SD	Standard Deviation
LED	Light-Emitting Diode
MPa	Megapascal
MDP	10-Methacryloyloxydecyl dihydrogen phosphate
